# Urinary Extracellular Vesicles for Diabetic Kidney Disease Diagnosis

**DOI:** 10.3390/jcm10102046

**Published:** 2021-05-11

**Authors:** Goren Saenz-Pipaon, Saioa Echeverria, Josune Orbe, Carmen Roncal

**Affiliations:** 1 Laboratory of Atherothrombosis, Program of Cardiovascular Diseases, Cima Universidad de Navarra, 31008 Pamplona, Spain; gsaenzdepip@alumni.unav.es (G.S.-P.); josuneor@unav.es (J.O.); 2 IdiSNA, Instituto de Investigación Sanitaria de Navarra, 31008 Pamplona, Spain; 3 Endocrinology Service, Clínica Universidad de Navarra, 31008 Pamplona, Spain; secheverriaa@unav.es; 4 CIBERCV, Instituto de Salud Carlos III, 28029 Madrid, Spain

**Keywords:** diabetes mellitus, diabetic kidney disease, nephropathy, urinary extracellular vesicles, biomarkers

## Abstract

Diabetic kidney disease (DKD) is the leading cause of end stage renal disease (ESRD) in developed countries, affecting more than 40% of diabetes mellitus (DM) patients. DKD pathogenesis is multifactorial leading to a clinical presentation characterized by proteinuria, hypertension, and a gradual reduction in kidney function, accompanied by a high incidence of cardiovascular (CV) events and mortality. Unlike other diabetes-related complications, DKD prevalence has failed to decline over the past 30 years, becoming a growing socioeconomic burden. Treatments controlling glucose levels, albuminuria and blood pressure may slow down DKD evolution and reduce CV events, but are not able to completely halt its progression. Moreover, one in five patients with diabetes develop DKD in the absence of albuminuria, and in others nephropathy goes unrecognized at the time of diagnosis, urging to find novel noninvasive and more precise early diagnosis and prognosis biomarkers and therapeutic targets for these patient subgroups. Extracellular vesicles (EVs), especially urinary (u)EVs, have emerged as an alternative for this purpose, as changes in their numbers and composition have been reported in clinical conditions involving DM and renal diseases. In this review, we will summarize the current knowledge on the role of (u)EVs in DKD.

## 1. Introduction

Type 2 diabetes mellitus (T2DM) is a global epidemic with an increasing prevalence worldwide, affecting 8.8% of adults (415 million people) [1]. In the last two decades, diabetes-associated deaths rose by 70%, making it a growing socioeconomic concern. Moreover, hyperglycemia-related morbi-mortality is very frequently linked to the development of complications affecting organ systems such as the kidney, which might lead to diabetic kidney disease (DKD), representing the main cause of end-stage renal disease (ESRD) in developed countries, affecting more than 40% of DM patients [1,2,3].

DKD is defined as the presence of altered kidney function in diabetic patients, diagnosed by an estimated glomerular filtration rate (eGFR) <60 mL/min/1.73 m^2^ and/or increased urinary albumin excretion (≥30 mg/g creatinine) persisting for >3 months, provided that other causes of chronic kidney disease (CKD) are excluded [4,5].

The pathogenesis of DKD is multifactorial and contributes to the progressive decline in the glomerular filtration rate, affecting tubuloglomerular feedback and inducing tubule hypertrophy, podocyte injury, albuminuria, inflammation, endothelial dysfunction, fibrosis, etc. [6]. As a consequence, hormonal and hemodynamic changes (including microcirculation impairments) are produced, and circulating levels of advanced glycation end products (AGEs), inflammatory mediators and/or growth factors increase [4]. This leads to a clinical presentation characterized by proteinuria, hypertension, and progressive reduction in kidney function [7].

Hyperglycemia and hypertension are the most important factors contributing to the progression of DKD [5]. Intensive glycemic control reduces the incidence of albuminuria by 50% and normotensive patients with advanced DKD show slower progression of kidney disease than do patients with hypertension. Accordingly, the primary strategy for preventing the development of DKD should aim at maintaining HbA1c <6.5% and blood pressure <140/80 mmHg [6,8].

There is an established relationship between albuminuria and cardiovascular (CV) disease, and as a consequence microalbuminuria and DM are considered risk factors for CV pathologies [4]. Therefore, interventions to decrease albuminuria and an intensive glycemic control have a positive effect on CV protection. The problem remains on those patients with DKD with high levels of albuminuria, where the normalization of the blood glucose might not completely halt the progression of the pathology. Moreover, almost 20% of patients with diabetes develop DKD in the absence of albuminuria [6], and in many others the coexistence of T2DM and nephropathy at the time of diagnosis can go unrecognized for years due to the complex natural history of T2DM [7].

The growing incidence of CV morbi-mortality in patients with DKD and the lack of precise biomarkers, other than albuminuria, for outcome assessment in this pathology reveals the need for: (1) novel noninvasive early diagnosis and prognosis candidates for the identification of patients with T2DM with high probability of DKD development and progression, and (2) alternative therapeutic targets to treat these high-risk patients. Extracellular vesicles (EVs) have emerged as useful noninvasive alternatives for this purpose, as changes in their numbers and composition have been reported in clinical conditions involving DM and renal diseases [9,10]. EVs are lipidic nanospheres actively released to the extracellular space by most cell types, that carry proteins, nucleic acids or metabolites from parental cells participating in cell-to-cell communication processes [11]. Renal EVs have been found in various body fluids including plasma and urine. The latter presents an additional advantage for EVs studies on metabolic and kidney diseases, considering that urinary EVs, (u)EVs, seem to be mainly of renal origin [12]. Consequently, the analysis of uEVs might mirror the functional and morphological changes suffered by the kidney in medical conditions such as DKD.

In this review, we will give insights into EVs’ biology, and the methods for their separation and characterization from urine and blood. Then, we will summarize the role of renal cell-derived EVs in cellular communication and explore the value of uEVs and circulating EVs as biomarkers, molecular effectors and/or possible therapeutic targets in DKD.

## 2. Extracellular Vesicles

The term EVs comprises acellular nanoscale particles delimited by a lipid bilayer, heterogeneous in size, that cannot replicate [13]. They are released habitually by most cell types in both physiological and pathological conditions, contributing to maintain tissue homeostasis, but also regulating complex processes during disease development. EVs contain nucleic acids, mainly mRNAs and noncoding RNAs, lipids, proteins and metabolites from the cell of origin. First considered cellular debris, currently the role of EVs in cell-to-cell communication is increasingly being recognized [14,15]. Traditionally EVs have been classified into three groups based on their size and biogenesis, distinguished as (a) exosomes, generated by the inward budding of the endosomal membrane, which are released to the extracellular space after the fusion of mature multivesicular endosomes with the cell membrane and present diameters from 30 to 150 nm [16]; (b) microvesicles, bigger in size (100–1000 nm), which are originated by the outward budding of the plasma membrane. They carry surface-specific antigens from the parental cell and in most cases also phosphatidylserine on the outer membrane leaflet [17,18,19]; and (c) apoptotic bodies, generated in the late steps of apoptosis, which present diameters between 1000–5000 nm, and can eventually contain organelles or nuclear fragments from the parental cells [13,20]. Given the overlap in size distribution, density and/or composition among different EV subpopulations, particularly exosomes and microvesicles, and the lack of suitable technologies to discriminate their subtypes once released to the extracellular medium, the International Society for Extracellular Vesicles (ISEV) coined the term EVs to encompass all vesicular subpopulations. Consequently, they encourage the individualized definition of EVs for each study based on their size, density, biochemical composition or cellular origin [13,21]. In this review, the term EVs will refer to both exosomes and microvesicles.

The interest in EVs as biomarkers and biological effectors might be attributed in part to their cargo, that it is protected from extra-vesicular nuclease and protease-mediated degradation by the lipid bilayer, which additionally gives them a stable structure for long-term storage and repeated freezing–thawing cycles [22]. EV content varies according to the cell or organ of origin and the microenvironment at the time of their generation, determining in this manner their fate and biological activity [22]. Thus, EV content could be considered a restricted, but very informative, snapshot of the molecular changes triggered by ongoing homeostatic or pathophysiological processes in parental cells at the time of their release. Moreover, medium-sized EVs or microvesicles directly shed from the plasma membrane can harbor molecular markers specific to the cell of origin, enabling the identification of EV subpopulations by cellular origin [18,23]. EV cargo analysis will greatly benefit from the current technological advances, including high-sensitivity nanoflow cytometers for EV phenotyping or adapted high-throughput technologies for low protein or nucleic acid inputs [22]. This might help to identify novel molecular biomarkers or therapeutic targets in diverse pathological conditions including metabolic and renal diseases.

EVs are able to interact with neighboring and/or distant cells transferring their cargo, rich in proteins, lipids or nucleic acids from parental cells to recipient cells, and thus participate in cell-to-cell communication processes. Although incompletely understood, it seems that EVs can be internalized by host cells through different mechanisms including pinocytosis, endocytosis, phagocytosis, or by direct fusion with the plasma membrane delivering their content into the cytosol, and modifying the physiological state of the host cell [14,15,20,24,25,26,27,28,29,30,31,32,33]. EVs can also stimulate specific signaling pathways via receptor–ligand interactions on the surface of the recipient cell, and additionally they are capable of inducing direct biological effects depending on their surface components. For instance, EVs rich in proteases or phosphatidylserine in the outer membrane leaflet can potentially degrade extracellular matrix components or bind coagulation factors contributing to tissue remodeling and thrombosis respectively [34,35].

One attractive particularity of EVs consists of their presence in all body fluids, including blood, ascites, cerebrospinal fluid, saliva, milk, and urine; blood still remaining the preferred source for EVs analysis. However, in renal and metabolic diseases, urine stands out as a sound alternative for EV studies [12,36]. First, urine collection is noninvasive, fast and easy, and second, it has been postulated that uEVs, coming from all parts of the nephrons and collecting ducts, might mainly be of renal origin, since circulating EVs are not able to pass through the glomerular membrane, at least in physiological conditions [10]. Therefore, the study of uEV content and distribution might give insights into the physiopathology of DKD, and contribute to the discovery of novel early diagnosis and risk stratification biomarkers, as well as therapeutic targets in this pathology. Nevertheless, as explained in the following section, prior to sample collection and EVs analysis some shortcomings of current uEV and EV separation and characterization techniques should be considered.

## 3. Challenges in uEV and EV Separation and Characterization

EVs can be isolated from multiple biological sources, including conditioned medium, biofluids (liquid biopsy) or tissues [37,38]. Urine and plasma, commonly used in EV studies, are complex biofluids containing large amounts of nonvesicular contaminants that hamper the efficient isolation of EVs. For instance, the Tamm Horsfall protein (THP), also known as uromodulin, is an abundant protein in urine that leads to uEV entrapment and precipitation thus, reducing final uEV yield. In blood, however, the lipoproteins HDL, LDL, VLDL, and chylomicrons presenting similar size and/or density to that of the circulating EVs constitute some of the main contaminants and technical challenges related their isolation [39].

Sample retrieval, preprocessing and storage conditions are critical for preserving the native morphological and molecular properties of EVs before their isolation, characterization and downstream analysis [13]. Regarding urine sample retrieval, spot urine (e.g., first morning void) or 24-h samples are commonly collected, and protease inhibitors might be added to improve specimen preservation [40]. After removing cells and debris by low-speed centrifugation (500–2000× *g*), cell-free urine samples can be stored at −80 °C for long-term storage without altering sample properties. Extensive vortexing of the frozen samples is recommended to improve uEV recovery after thawing [41]. Additionally, measuring urine creatine levels is commonly used to estimate uEV concentration in the sample, enabling normalization of uEV input and subsequent comparison between individuals. Other methods such as Nanoparticle Tracking Analysis (NTA) are also used for measuring uEV concentration, although particle levels might be overestimated in patients with albuminuria. Normalization to THP or EV-related proteins levels (e.g., TSG-101 or Alix) has also been suggested, assuming that their expression is maintained constant in different pathophysiological conditions [42].

Regarding retrieval of blood, overnight fasting samples are preferentially used to reduce lipoprotein contamination. Special care should be taken to avoid platelet activation due to clot formation, thus blood is collected using large diameter (≥21-gauge) needles that reduce shear stress, discarding the first 2–3 mL of blood [43]. Citrated plasma is the most commonly used anticoagulant for EV studies, although others such as acid-citrate dextrose have shown lower generation of platelet EVs [44]. Just after collection, samples are gently mixed and kept at room temperature, avoiding agitation. Hemolysis should be checked by visual inspection or spectrophotometric methods and hemolyzed samples preferably discarded to prevent biased molecular analyses. Blood samples are then processed by two sequential low-speed centrifugations (500× *g* for 15 min) obtaining platelet-free plasma and snap frozen in liquid nitrogen for long-term storage at −80 °C [45].

When separating EVs, ultracentrifugation (UC) is the classical and still most commonly used method for isolating both EVs and uEVs based on their density [46]. Cell-free urine or platelet-free plasma samples undergo subsequent high-speed centrifugation steps at different speeds; 10,000–20,000× *g* or ~100,000× *g* to pellet medium/large or small size EVs respectively. Despite its simplicity, UC requires high amount of starting material and contaminating protein aggregates are pelleted together with EVs. Treatment of urinary samples with dithiothreitol (DTT) or 3-[(3-cholamidopropyl)dimethylammonio]-1-propanesulfonate (CHAPS) detergent is recommended to release EVs from THP aggregates [47,48]. Combining UC with other methods such as density gradient UC using sucrose or iodixanol (Optiprep™) notably reduces protein contamination, although EV yield might be compromised. Additionally, both UC and density gradient UC are time-consuming and low-throughput methods, which represents a major drawback for their applicability in clinical scenario.

Size exclusion chromatography (SEC) represents a valuable method for the rapid isolation (~15 min) of urinary or blood EVs, efficiently enriching and separating them from contaminants such as THP or lipoproteins, respectively. SEC is commonly combined with ultrafiltration (UF) to further concentrate the purified EVs [42]. UF techniques enable rapid isolation and concentration of EVs using nanomembranes with a suitable size cut-off of ~100 kDa. However, some EV populations might adhere to the nanomembranes and abundant proteins such as albumin might obstruct the nanopores, compromising EV yield and purity when isolating uEVs and EVs from proteinuric patients or blood samples [49,50]. Alternatively, hydrostatic filtration dialysis (HFD) using 1000 kDa dialysis membranes has been shown to be a suitable method for isolating and concentrating uEVs from large sample amounts, 1–2 L to 2–3 mL, facilitating sample storage and handling while maintaining high uEV yield and purity [51].

Precipitation methods consistent of polymer-based mixtures such as polyethylene glycol (PEG), represent an effective way for concentrating uEVs and EVs but not for removing protein aggregates. Several commercial kits are currently available for EVs precipitation from different biofluids, including ExoQuick™ (System Biosciences) or miRCURY™ Exosome kit (Qiagen). Affinity-based techniques using antibodies against specific EVs surface markers are also commonly used for purifying particular EV subpopulations. Typically, magnetic bead-bound antibodies are used to separate EVs using magnets. Additionally, other molecules such as proteins or synthetic peptides can also be used to bind common EV surface markers (e.g., phosphatidylserine or heat shock proteins) allowing their isolation with high yield and purity [52,53].

To overcome the major drawbacks of current EV and uEV separation methods, several novel procedures are being developed, including microfluidic-based devices, flow field-flow fractionation, or high-resolution flow cytometry [37]. For instance, ExoDisc™ (LabSpinner) microfluidic tangential flow filtration device has recently been shown to efficiently isolate uEVs, presenting higher uEV recovery than UC, precipitation or SEC followed by UF. Although protein contamination was slightly higher in the microfluidic device compared to SEC+UF, both methods showed complete removal of THP in Western-blot analysis. Additionally, ExoDisc™ required less than 30 min for completion, indicating its suitability for clinical scenario [54].

Once EVs are isolated, their purity, concentration and morphology should be characterized using different complementary techniques. For assessing EV purity and the presence of nonEV contaminants, Western blotting (WB) is the preferred method. Specific EV surface markers (e.g., CD63, CD81 or Alix) and abundant contaminants of urine (e.g., THP) and plasma/serum (e.g., lipoprotein and albumin) can be readily detected by this technique. NTA is commonly used to determine both size and concentration of single particles, being able to analyze EVs between 50 and 1000 nm [55]. High-resolution flow cytometry is also used for single EV analysis and may be combined with specific EV surface markers enabling quantification of EV subpopulations derived from podocytes (e.g., podocin), proximal tubular epithelium cells (e.g., megalin), platelets (e.g., CD41/61) or erythrocytes (e.g., CD235a), among others [46,56]. In addition, EVs can be labeled with nonfluorescent pro-dyes such as calcein or 5-(and-6)-Carboxyfluorescein diacetate N-succinimidyl ester (CFSE), which are processed by intracytoplasmic enzymes, resulting in impermeant fluorescent molecules [57]. Imaging techniques such as conventional transmission electron microscopy (TEM) and cryo-TEM, which minimizes changes in EV morphology, are useful for visualizing EVs and detecting of nonEV particles, including THP and lipoproteins. Additionally, EV surface epitopes can also be detected by immunogold labeling using either TEM or cryo-TEM [58]. Other downstream analysis techniques such as transcriptomics or proteomics are currently being used to further depict the heterogenous content of EVs and might help to further standardize EV characterization process, although their low nucleic acid and protein content still represents a challenge for current high-throughput technologies.

In sum, current separation procedures do not allow an absolute purification of either uEVs or EVs from other nonvesicular contaminants that overlap in size and density [13]. Thus, EV isolation methods are often selected based on their downstream application, such as in the case of biomarker discovery, where EV purity is commonly preferred to high yield. Additionally, sample retrieval and storage conditions, as well as EV separation methods, should be thoroughly described to enable reproducibility, and a complete description of the complementary characterization techniques applied to assess purity, integrity and concentration of the isolated EVs should be also included.

## 4. Biological Effects of EVs on Renal Cells

Metabolic alterations might affect the release of EVs from kidney cells, affecting their numbers, but also their cargo, which is rich in proteins, lipids, metabolites, mRNAs and mi(cro)RNAs from the parental cell. As such, those EVs generated in response to glomerular or tubular injury might be able to transfer survival and/or damage signals to neighboring or distant cells, promoting diverse biological effects. Several authors have studied the impact of metabolic stimuli, mainly high glucose (HG), in EVs release by glomerular [59,60,61,62,63,64] and tubular cells [65], and in their associated biological activities (Table 1).

Podocytes are essential for the maintenance of the glomerular filtration barrier (GFB) and diabetes-induced podocytopathy, resulting in increased glomerular permeability and posterior albuminuria, is considered a key event in the initiation of DKD [74]. Despite this precedent, little is known about the early molecular modifications induced by hyperglycemia in podocytes. In this regard, the effect of HG in EVs, either in their numbers or composition, might reflect early podocyte damage (Table 1). In vitro, HG elicited the generation of Wilm’s tumor-1 (WT1) mRNA-enriched podocyte EVs [66], a urinary protein associated with decreased kidney function in DKD [75]. Based on these results the authors proposed that the determination of WT1 mRNA in podocyte EVs could be an alternative to current methods for WT1 quantification in urine, often masked by albuminuria in renal diseases [66]. Other authors have reported the possible involvement of reactive oxygen species (ROS) and NADPH Oxidase (NOX)4 in the processes leading to podocyte EV release in presence of HG [60]. Interestingly, podocyte EVs have been shown to regulate proximal tubular epithelial cells (PTECs) function in vitro. In particular, HG-podocyte EVs induced PTEC dedifferentiation through miRNA-221 and Wnt/β-catenin signaling [67], and the activation of fibrotic responses by increasing the expression of fibronectin, collagen type IV, p38, and the phosphorylation of Smad3 [68]. In addition, other authors observed PTECs apoptosis in response to HG-podocyte-EVs in a mechanism related to the differential encapsulation of 5 miRNAs [59]. These data suggest a possible role of podocytes in proximal tubule cell injury in a process involving EV release as reservoirs of bioactive molecules.

Glomerular endothelial cells (GECs) are the first layer of the GFB and contribute to maintaining glomerular homeostasis in response to hemodynamic changes, conforming the first boundary to macromolecular substances. Consequently, GEC dysfunction results in the impairment of the GFB and the appearance of albuminuria, representing an early and deleterious hallmark of DKD [74]. The study of GECs-EVs offers an opportunity to gain insight into the mechanisms triggered by HG in glomerular endothelium, but also to unravel their interaction with other renal cells. For instance, in response to HG in vitro, GECs released EVs enriched with the profibrotic protein TGF-β [61,62], which in turn promoted podocyte epithelial–mesenchymal transition [61] and glomerular mesangial cell activation in a TGF-β1-dependent manner [62]. In vivo, the systemic administration of HG-GECs-EVs induced mesangial expansion, proliferation and extracellular matrix protein overproduction in kidney tissues of WT mice [62], supporting a role of GEC-EVs in glomerular dysfunction.

Glomerular mesangial cells (GMCs) have been also investigated as sources and targets of EVs. Indeed, GMC-derived EVs were similar in size, but not in number, in HG-stimulated cultures compared to controls, exerting different biological effects. As such, the co-incubation of GMCs with mesangial HG-EVs resulted in the production of higher levels of fibronectin, angiotensinogen, renin, Angiotensin II type (AT)1 and AT2 receptors in GMCs, indicating their possible role in kidney cell dysfunction [64]. In contrast, Barutta et al. reported a reduction in the number of GMC-EVs by HG compared to control cultures, which were enriched in miR-145 [63], while others found no differences in miR-192, -194 or -215 despite HG treatment [70]. Wang YY et al. showed that the coincubation of podocytes with HG-GMCs-EVs resulted in podocyte apoptosis and impaired cellular adhesion, which was reverted when TGF-β was blocked either by siRNA or a chemical treatment with berberine [69]. The studies summarized above point towards a crosstalk between all glomerular components via EVs, prominently through oxidative stress and fibrosis-related processes.

Initially, diabetes affects the glomerulus, and later promotes tubular hypertrophy, fibrosis, inflammation and renal function impairment [74]. EVs have been postulated as possible vehicles by which damaged podocytes could transfer detrimental signals to tubular epithelial cells [60,68,69]. In addition, other authors have shown that HG-PTCs-EVs activate downstream molecular targets including constituents of the TGF-β, mTOR, ERK and endoplasmic reticulum stress pathways on naïve PTCs in vitro [71], suggesting that pharmacological interventions to inhibit the shedding of EVs from PTCs might serve as an effective strategy for preventing the progression of DKD [71]. Moreover, the tubular epithelial cell line HK-2, when exposed to HG, released EVs enriched in miR-192, -194 and -215, indicating that the biological changes induced by tubular cell-EVs could be mediated by variations in the sorted miRNAs [70]. Finally, HG-tubular-EVs induced higher proliferation and fibrosis marker expression in cultured renal fibroblast, suggesting a role for EVs in cell-to-cell communication processes between tubular cells and neighboring fibroblasts [65].

Despite the suspected involvement of inflammation, particularly macrophage infiltration, in DKD progression, the exact biological and molecular mechanisms governing this process are still unknown, although some evidence support the participation of EVs. In this regard, the systemic administration of EVs isolated from HG-exposed macrophages resulted in pathologic glomerular remodeling in WT mice, inducing renal expression of molecules related to inflammation (iNOS, IL-1β) and fibrosis (TGF-β1, α-SMA, collagen IV and fibronectin) through the activation of the NF-κB/p65, and the TGF-β/Smad3 signaling pathways, respectively [72,73].

Overall, the experimental evidence supports an active and dynamic role of EVs in intercellular communication between glomerular and tubular cells in response to diabetes. Despite this very exciting perspective, deeper and more extensive in vitro and in vivo analysis is needed to clarify the exact changes induced by diabetic stimuli in EVs cargo and release. This will also permit a more insightful understanding of the processes leading to EV–cell communication and the resulting (patho)physiological mechanisms. Moreover, the identification of specific EV subpopulations in relation to their origin, specific proteins, or RNA content might be useful in the search of novel biomarkers and therapeutic targets in DKD.

## 5. Urinary Extracellular Vesicles (uEVs) as Potential Biomarkers in Diabetic Kidney Disease

DKD diagnosis has traditionally been based on microalbuminuria; however, renal structural damage might precede albumin excretion, as suggested by the number of diabetic patients (about 20%) that develop DKD in the absence of albuminuria [6], limiting the accuracy of its diagnosis in a significant part of DM population. The lack of sensitivity and specificity of albumin for the early identification of high-risk patients has prompted the search for novel noninvasive alternatives to cover this clinical need. EVs, and more precisely uEVs, have emerged as a plausible option in this regard, since the structure of the glomerular membrane, at least in healthy condition, restrains the passage of circulating EVs to urine, and thus uEVs derived from all parts of the nephrons and collecting ducts might be mainly of renal origin [10]. Likewise, the determination of the total uEV numbers or specific uEV subpopulations, and the analysis of their content might help to identify novel biomarkers and therapeutic targets for DKD risk assessment in every segment of the nephron, serving as early biomarkers of renal dysfunction and structural injury (Table 2).

Early studies by Raimondo F et al. compared the proteomic profile of urine and uEVs from Zucker diabetic fatty rats as a model of T2DM. The proteomic analysis revealed that despite advanced proteinuria, uEVs presented a completely different protein pattern compared to urine, suggesting that uEVs content might represent glomerular and/or tubular cellular changes more faithfully than the whole urinary proteome [76]. The majority of identified proteins were membrane-associated or cytoplasmic and involved in transport, signaling and cellular adhesion, typical functions of EV proteins. According to these criteria, the content of 76 protein species out of 286 increased in diabetic EVs, while 68 decreased [76]. In humans, the application of proteomic methods to uEVs identified 22 differentially expressed proteins among diabetic patients with different degrees of renal impairment (Table 2). Four of them, Mannan-binding lectin serine protease 2 (MASP2) Calbindin (CALB1), S100A8 and S100A9, were selected as potential biomarkers of early DKD based on bioinformatic analysis, although no validation of the free or EV-encapsulated proteins was performed in either urine or blood (Table 2) [77]. Other authors have investigated the protease and protease inhibitor profile of uEVs in T1DM patients, describing distinctive alterations in protease profiles according to the levels of albuminuria [78]. Interestingly, myeloblastin and its natural inhibitor elafin showed an increase in the normo- and microalbuminuric groups. Similarly, a characteristic pattern was observed in the array of protease inhibitors, with a marked increase of cystatin B, natural inhibitors of cathepsins L, H, and B, as well as of neutrophil gelatinase-associated Lipocalin (NGAL) in the normoalbuminuric group [78]. In this line, Ning J et al. described no expression of α1- antitrypsin (AT) in uEVs of healthy or prediabetic patients, while uEV α1-AT content gradually increased in diabetic patients according to the aggravation of DKD and the decline of renal function, suggesting its possible use as an early diagnosis biomarker for DKD assessment [79]. Abe H et al. failed to detect WT1 protein in uEVs, but instead determined its mRNA levels, finding increased WT1 expression in uEVs of DN patients, which made it possible to discriminate DKD diagnosis by receiver operating characteristic (ROC) curves. Moreover, the progression to ESRD was lesser in patients with lower expression of WT1 mRNA in uEVs, suggesting that the determination of this specific uEV subpopulation might be meaningful for evaluating the susceptibility of DKD progression [66]. As such, of the eight candidate mRNAs (uromodulin, SLC12A1, NDUFB2, OAZ1, PPARGC1A, NFE2L2, CD24 and SMAD1) measured in uEVs of DKD, chronic kidney disease, T2DM, nondiabetic obese, and healthy controls, uromodulin was the most expressed transcript in mild and severe DKD, and significantly increased when compared to healthy controls [80].

Podocyte injury is an early hallmark of glomerular dysfunction, and consequently, any strategy enabling its prompt identification might be of great advantage for the classification of diabetic patients at risk of DKD. In this regard, Burguer D et al. determined by flow cytometry the levels of podocyte-derived uEVs in three models of T1DM and a fourth model of T2DM. Remarkably, the levels of podocalyxin+uEVs, a specific marker of podocytes, increased even before albuminuria was developed, suggesting that the elevation in podocyte-derived uEVs might be a sensitive and early indicator of podocyte damage in diabetes [91]. Similarly, a later report by Lytvyn Y et al. described increased levels of podoplanin+uEVs, another podocyte marker, in patients with T1DM compared with healthy controls, which further increased under hyperglycemic clamp [81]. Based on in vitro studies, where the treatment of podocytes with AGEs induced the secretion of EVs enriched in an upstream target of TGF-β signaling pathway, the epithelium-specific transcription factor-3 (Elf3), the levels of Elf3+uEVs were evaluated as markers of podocyte injury in patients with T2DM and heavy proteinuria. The authors reported an association of Elf3+uEVs with a decline in eGFR in DKD patients, while Elf3+uEVs were not detected in healthy subjects, suggesting the usefulness of this specific uEV subpopulation as an early noninvasive marker for podocyte injury in DKD [82].

Further studies have been focused on the utility of uEV protein content to assess renal tubular alterations (Table 2). As such, similar protein levels of aquaporin 1, a marker of PTECs, was observed in uEVs from healthy subjects, diabetics without evidence of renal injury and DKD patients, while CD73, an early target of the profibrotic TGF-β cascade, was enriched in uEVs of patients with nephropathy as compared with the other groups, suggesting its possible use to evaluate diabetic renal tubular alterations before eGFR declines [83]. Other authors found an association between C-megalin uEVs levels and the progression of the albuminuric stages in patients with T2DM [84]. Similarly, in a mouse model of obesity-related diabetes, C-megalin+uEVs levels increased in animals fed on a high-fat diet compared to chow diet, and in vitro, the release of C-megalin+EVs by proximal tubule cells increased after fatty acid-free bovine serum albumin (BSA) or AGE-modified BSA stimulation [84].

The presence of renal regenerative markers, such as CD133, a protein expressed by renal progenitor cells, has been also explored for DKD risk estimation. The numbers of CD133+uEVs decreased gradually as DKD progresses according to micro- or macroalbuminuria. Moreover, the ability of CD133+uEVs to discriminate the healthy condition from that of glomerular disease was corroborated by ROC curves [85]. In vitro, albumin reduced the content of the CD133 marker in renal progenitor cell-derived EVs, which was further decreased by the coincubation with both, albumin and HG [85].

Finally, several research groups have investigated the content and fluctuations of miRNAs in uEVs as potential biomarkers and regulators of DKD progression (Table 2). RNA sequencing found differential miRNA profiles in urine and uEVs in T1DM patients, uEVs fraction being enriched in the number of detected miRNAs [86]. Moreover, these authors identified specific miRNA signatures in uEVs according to the progression of nephropathy in patients developing overt, intermittent or persistent microalbuminuria during the follow up [86]. In this line, the levels of specific miRNAs: miR-145, miR-192, miR-194, miR-215, let-7i-3p, miR-24-3p, miR-27b-3p, miR-362-3p, miR-877-3p, miR-150-5p, miR-21-5p, and miR-26a were reported to be increased in association with DKD progression, while miR-15b-5p and miR-30b-5p decreased [63,70,87,88,89,90]. Moreover, by bioinformatic pathway analysis let-7i-3p, miR-24-3p, miR-27b-3p and miR-15b-5p were predicted to target protein networks involved in the Wnt/b-catenin signaling cascade, activin receptor signaling and cell differentiation/proliferation [87], while miR-362-3p, miR-877-3p, and miR-150-5p were associated with p53, mTOR, and AMPK pathways [88]. Considering the lack of standardization for uEV separation, Park S et al. compared the miRNA profile of uEVs from DKD patients using three different isolation methods. Despite differences in uEV yield, they found 22 overlapping miRNAs with similar expression levels among the tested purification techniques [92]. Additional experiments aiming to compare miRNA profiles between uEVs and circulating EVs, however, rendered variable correlations depending on the specific patients, suggesting different miRNA cargo in response to diabetes in urine and blood [92]. In addition, some authors have explored the biological role of some of those identified miRNAs in animal and cellular models. For instance, Zheng et al. observed that the stimulation of renal TPECs with TGFβ 1 increased the release of EVs enriched in miR-26a [90], and Barutta F et al. reported that mesangial cells in culture released EVs enriched in miRNA-145 in response to HG. In vivo miR-145 was expressed in the glomerular compartment of diabetic mice, that concomitantly presented a 2-fold increase in miR-145+uEVs compared to nondiabetic mice [63], suggesting a possible role of miRNA-145 as a biomarker, but also as a regulator in diabetes related DKD.

Altogether, current evidence suggests that uEVs might reflect more accurately the early changes induced by diabetic stimuli in glomerular and tubular cells as compared to the current gold standard in clinical practice, albuminuria. Even if promising, larger studies will need to be performed to clarify the usefulness of uEV determination for DM-associated DKD, as well as the molecular markers or molecules (protein, mRNAs or miRNAs) that best associate with disease phenotypes. Moreover, the implementation of uEVs analysis in clinical practice will greatly depend upon the simplification of EVs isolation and characterization techniques.

## 6. Circulating EVs in Diabetic Kidney Disease

Circulating EVs, although to a lesser extent than uEVs, have been also investigated as biomarkers for DKD diagnosis. In this regard, increased levels of platelet-, erythrocyte-, leukocyte-, and endothelial-derived EVs have been reported in DKD patients compared to controls [93,94,95]. The role of platelet-derived EVs in GEC dysfunction was also investigated in an experimental model of streptozotocin-induced diabetes in rats by Zhang Y et al. [96]. They described a gradual increase of platelet-derived (P)EVs in plasma of diabetic rats compared to controls, which in tissue was localized within the glomerular endothelium. Interestingly, aspirin treatment decreased the circulating levels of PEVs and, locally, lessened GEC damage. In vitro, PEVs isolated from diabetic rat plasmas or whole blood increased ROS production and decreased nitric oxide levels, promoting GECs permeability and a reduction in the endothelial surface layer, in a mechanism mediated by CXCL7/mTORC1 pathway [96]. Circulating medium-sized EVs or microparticles have traditionally been assigned a procoagulant activity regarding their cargo in phosphatidylserine (PS) and coagulation factors (e.g., tissue factor, TF) and have been studied in relation to DKD-associated hypercoagulability. As such, the levels of TF and PS positive EVs increased in DKD patients compared to controls according to albuminuria [93,94]. Moreover, in vitro, the procoagulant activity of isolated EVs, assessed by recalcification-time assays, showed decreased coagulation time consistent with DKD severity [94].

miRNA content and the function of circulating EVs has also been studied in DKD. In this respect, Florijn BW et al. reported increased levels of miRNA-21 and -126 in plasma EVs of DKD patients compared to EVs of healthy controls or DM patients without DKD. In vitro, EVs rich in miR-21 improved endothelial barrier formation of cells cultured in serum from patients with DM and DKD [97]. Similar studies by Uil M et al. described the upregulation of miR-99a-5p in EVs of macroalbuminuric patients compared with normo- and microalbuminuric subjects [95]. Moreover, the transfection of the miR-99a-5p mimic to cultured podocytes induced a downregulation of mTOR and the injury marker vimentin, suggesting a protective effect of miR-99a-5p on glomerular cells [95]. Sequencing of circulating EVs found a different miRNA profile in healthy volunteers compared to diabetic patients with DKD [98]. Likewise, the eight differentially expressed miRNAs in DKD (miR-1246, miR-642a-3p, let-7c-5p, miR-1255b-5p, let-7i-3p, miR-5010-5p, miR-150-3p and miR-4449) were significantly correlated with the degree of albuminuria, and were involved in MAPK signaling, angiogenesis, and regulation of the AP-1 transcription factor when pathway analysis was performed [98].

Data regarding the role of blood EVs in DKD are scarce and include a relatively low number of patients, although from the available data, it could be speculated that specific subpopulations of EVs, particularly those derived from platelets or those carrying procoagulant proteins, might be potential biomarkers and/or biological effectors in DKD. In this regard, the regulatory role of the miRNAs encapsulated by circulating EVs should also be considered.

## 7. Conclusions/Future Directions

Epidemiological data predict a great increase in metabolic and renal diseases as a consequence of lifestyle changes and aging. At present, available clinical diagnosis tests lack sensitivity and specificity for the early and accurate classification of diabetic individuals at risk of developing DKD. EVs and, more particularly, uEVs have emerged as promising alternatives in this regard. As such, the data summarized in this review suggest that EVs might participate in DKD development and progression by regulating molecular pathways related to fibrosis, either by carrying fibrosis-related molecules or by inducing the expression or the activation of different fibrosis associated pathways (e.g., TGF-β/smad3, Wnt/β-catenin or mTOR) in target cells. Besides their function as biological effectors, the concentration and subtypes of uEVs, for example those enriched in TGF-β or WT1, have also been proposed as early diagnosis biomarkers of renal dysfunction in DM. Although very promising, these results will need to be confirmed in larger experimental and clinical studies. In addition, the combined analysis of urinary and blood EVs might render complementary information on the effect of diabetes in kidney cells and circulating and vascular components respectively, even before albuminuria is detectable. Moreover, in vitro and in vivo experiments support an active and dynamic role of EVs for intercellular communication between glomerular and tubular cells, and will be key in understanding the molecular mechanism activated in response to diabetes, which in the clinical setting may lead to DKD and, in the worst cases, progress to ESRD. The possibility of studying the proteomic and/or genomic content of (u)EVs in the early stages of DM or DKD might help to identify patients with molecular profiles at risk of worse progression, who will benefit from more personalized medical care in the future. It is worth considering, however, that the limitations of current (u)EVs separation and characterization procedures might delay the implementation of EVs analysis in the daily clinical practice.

## Figures and Tables

**Table 1:** In vitro and in vivo studies analyzing the functional role of EV on different renal cell types.

EV Source	Target Cells/ Organ	Study Type	Observation	Biological Activity	Ref.
Mouse podocytes	-	In vitro	High glucose (HG) increased Wilm’s tumor-1 (WT1) mRNA in podocyte-derived EVs.	Specific EV subpopulations as early podocyte injury biomarker	[66]
Mouse podocytes	-	In vitro	In HG conditions, release of podocyte EVs was reduced upon silencing of NOX4 pathway.	Characterization of EV release mechanisms	[60]
Human podocytes	PTECs	In vitro	HG-podocyte EVs, enriched in miR-221, induced PTECs dedifferentiation through Wnt/β-catenin signaling.	Intercellular communication. Dedifferentiation and fibrosis signaling activation in target cells	[67]
Human podocytes	PTECs	In vitro	Podocyte EVs increased the expression of fibronectin, collagen type IV, p38, and phosphorylated Smad3 in PTECs.	Intercellular communication. Fibrosis signaling activation in target cells	[68]
Mouse podocytes	PTECs	In vitro	HG-podocyte EVs induced apoptosis of PTECs and showed differential loading of miR-1981, -3474, -7224, -6538, and let-7f-2.	Intercellular communication. Transcriptional regulation through miRNA transport. Apoptosis signal transduction in target cells	[59]
Mouse glomerular endothelial cells (GECs)	Podocytes	In vitro	EVs from HG-GECs were enriched in TGF-β1 and promoted podocyte epithelial-mesenchymal transition (EMT) and dysfunction.	Intercellular communication. Induced podocyte EMT and dysfunction.	[61]
Mouse GECs	GMCs	In vitro In vivo	HG-GEC-EVs were enriched in TGF-β1 and induced mesangial expansion, GMC proliferation and ECM protein overproduction in vivo and in vitro.	Intercellular communication. Tissue remodeling. Induced proliferation and fibrosis in target cells.	[62]
Human GMCs	GMCs	In vitro	The exposure of GMCs to HG-GMC-EVs increased the expression of fibronectin, angiotensinogen, renin, AT1 and AT2 receptors.	Intercellular communication. Induced fibrosis activation in target cells	[64]
Human GMCs	-	In vitro	HG reduced the release of GMC-EVs but increased their miR-145 loading.	Transcriptional regulation through specific miRNA encapsulation	[63]
Rat primary GMCs	Podocytes	In vitro	HG-GMC-EVs impaired podocyte cell adhesion and promoted apoptosis via TGF-β1 signaling.	Intercellular communication. Induced fibrosis and apoptosis in target cells	[69]
Human PTEC and GMC	-	In vitro	HG increased the expression of miR-192, -194 and -215 in PTEC-EVs but not in GMCs-EVs.	Transcriptional regulation through specific miRNA encapsulation in PTECs	[70]
Rat PTC	PTCs	In vitro	HG-PTC-EVs activated TGF-β, mTOR, ERK and endoplasmic reticulum stress pathways in naïve PTCs.	Intercellular communication. Fibrosis signaling activation in PTCs	[71]
Mouse PTCs	Fibroblast	In vitro	HG reduced PTC-EV release. HG-PTC-EVs promoted fibroblast proliferation and protein expression of fibronectin, collagen type I and α-SMA.	Intercellular communication. Fibrosis signaling activation in fibroblast.	[65]
Mouse macrophages	GMCs C57BL/6 WT mice	In vitro In vivo	HG-macrophage-EVs were enriched in iNOS, IL-1β, and TGF-β1 and induced ECM production and inflammatory factor secretion from GMCs in vitro and in vivo through NF-κB/p65 and TGF-β1/SMAD3 signaling pathways.	Intercellular communication. Tissue remodeling. Inflammation and fibrosis activation	[72,73]

AT1 and 2 receptors: Angiotensin II type (AT)1 and AT2 receptors, α-SMA: alpha-smooths muscle actin, ECM: extracellular matrix, ERK: extracellular signal-regulated kinases, GECs: glomerular endothelial cells, GMCs: glomerular mesangial cells, HG: high glucose, iNOS: inducible nitric oxide synthase, IL-1β: interleukin-1beta, mTOR: mammalian target of rapamycin, NOX4: NADPH Oxidase 4, PT(E)Cs: proximal tubular (epithelial) cells, ROS: reactive oxygen species, TGF-β1: Transforming growth factor beta 1, WT1: Wilm’s tumor-1.

**Table 2:** Summary of studies analyzing the role of uEVs in DKD patients according to their protein and mRNA content, uEV subpopulations and miRNA.

Patient Groups (*n* = Number)	Observation	Application of uEVs	Refs.
*uEV protein and mRNA content*			
Healthy controls (*n* = 15), pre-DM (*n* = 15), diabetes with normal proteinuria (NA) levels (*n* = 15), DM with microalbuminuria (MIC, *n* = 15), and DM with macroproteinuria (MAC, *n* = 15)	Protein concentration was higher in uEVs of DM vs. controls. MASP2, CALB1, S100A8 and S100A9 identified as potential biomarkers of DKD by proteomics analysis.	uEV proteomics for the identification of novel biomarkers and/or therapeutic targets	[77]
Healthy volunteers (*n* = 12), T1DM with different degrees of albuminuria (*n* = 37)	Myeloblastin and elafin increased in the T1DM-NA and T1DM-MIC uEVs. Cystatin B, natural inhibitor of cathepsins L, H, and B, and NGAL increased in the T1DM-NA group.	uEV proteomics for the identification of novel biomarkers and/or therapeutic targets	[78]
Healthy people (*n* = 40), prediabetic patients (*n* = 40), diabetics with: NA (*n* = 28), MIC (*n* = 28) and MAC (*n* = 11)	No expression of α1- AT protein in uEVs of healthy or prediabetic patients. uEV α1-AT content gradually increased in diabetic patients according to DKD degree.	α1-AT+ uEVs; biomarkers of DKD severity	[79]
DKD with heavy proteinuria (*n* = 10), MCNS (*n* = 10), healthy subjects (*n* = 5)	WT1 mRNA expression increased in uEVs of DKD vs. controls. Low expression of WT1 in uEVs was associated with lesser progression to ESRD.	WT1+uEVs; biomarkers of DKD diagnosis and progression	[66]
Healthy (*n* = 18), obese (*n* = 18), T2DM (*n* = 161), mild DKD (*n* = 19) and severe DKD (*n* = 15)	Uromodulin mRNA in uEVs was elevated in DKD vs. healthy, obese, and T2DM subjects.	Uromodulin+uEVs; biomarkers of DKD diagnosis	[80]
*uEV subpopulations*			
Healthy controls (*n* = 20), T1DM (*n* = 25)	Podoplanin^+^uEVs were elevated in T1DM vs. control, and further increased after hyperglycemic clamp.	Podoplaning+uEVs; podocyte injury biomarkers	[81]
Healthy subjects (*n* = 5), DKD with heavy proteinuria (*n* = 25) or MCNS (*n* = 25)	Elf3+uEVs undetected in healthy subjects. Elf3+uEVs associated with a decline in eGFR in DKD.	Elf3+uEVs; podocyte injury and DKD severity biomarkers	[82]
Nondiabetic (*n* = 10), diabetic (*n* = 48) and DKD (*n* = 10)	CD73 was enriched in uEVs of DKD vs. control and diabetes.	CD73+uEVs; DKD diagnosis and tubular fibrosis biomarkers.	[83]
Controls (*n* = 19), T2DM-NA (*n* = 20); T2DM-MIC (*n* = 17); and T2DM-MAC (*n* = 19)	The levels of total uEVs and C-megalin^+^uEVs increased according to albuminuria in patients with T2DM.	C-megalin+uEVs; biomarkers of DKD diagnosis and tubular fibrosis	[84]
Controls (*n* = 13), T2DM-NA (*n* = 17), T2DM-MIC (*n* = 15) and T2DM-MAC (*n* = 15)	CD133^+^uEVs decreased in T2DM vs. control and within diabetic according to MIC or MAC.	CD133+EVs; biomarkers of tissue regeneration and DKD diagnosis and severity	[85]
*uEVs miRNA content*			
T1DM patients with a follow-up of 25 years that developed: overt nephropathy (*n* = 8), intermittent MIC (*n* = 9), persistent MIC (*n* = 10) and with no evolution (NA, *n* = 5)	uEVs of T1DM patients were enriched in miRNAs compared to urine. Overt patients presented 21 differential miRNAs in uEVs vs. NA	uEVs miRNA profile associated with DKD progression	[86]
Nondiabetic subjects (*n* = 10), and T1DM: T1DM-NA (*n* = 12), T1DM-MIC (*n* = 12)	uEVs were reduced in T1DM-MIC patients. miR-155 and miR-424 were lower, while miR-130a and miR-145 were higher in T1DM-MIC than in T1DM-NA patients.	uEVs miRNA profile associated with DKD severity	[63]
TD2M-NA (*n* = 30), T2DM-MIC (*n* = 30), T2DM-MAC (*n* = 20) and healthy controls (*n* = 10)	The levels of miRNA-192, -194 and -215 increases gradually among controls, NA, and MIC-T2DM, but decreases in the MAC group. uEVs content in TGF-𝛽1 correlated with that of miR-192 and -215.	uEVs miRNA profile associated with DKD severity and fibrosis	[70]
Subjects (*n* = 40 each) with normal glucose tolerance (NGT), T2DM-NA (*n* = 40), T2DM-MIC (*n* = 40) and T2DM-MAC (*n* = 40)	Let-7i-5p, miR-135b-5p, miR-15b-3p, miR-197-3p, miR-24-3p and miR-27b-3p discriminate T2DM-NA patients from those with T2DM-MIC and T2DM-MAC.	uEVs miRNA profile associated with DKD diagnosis	[87]
T2DM (*n* = 20) and T2DM-MAC (*n* = 20)	miR-362-3p, miR-877-3p, and miR-150-5p were upregulated and miR-15a-5p was downregulated in T2DM-MAC.	uEVs miRNA profile associated with DKD diagnosis	[88]
T2DM-DKD (*n* = 22), T2DM normal renal function (*n* = 15) and CKD without diabetes (*n* = 18)	miR-21-5p increased in uEVs of T2DM-DKD and CKD vs. T2DM, while miR-30b-5p was downregulated in both diabetic DKD and in CKD patients.	uEVs miRNA profile associated with DKD and CKD diagnosis	[89]
T2DM (*n* = 30) and T2DM-DKD (*n* = 20)	Expression of miR-26a was elevated in uEVs from DKD patients.	miRNA-26a associated with DKD diagnosis	[90]

α1-AT: alpha 1- antitrypsin, CKD: chronic kidney disease, DKD: diabetic kidney disease, ESRD: End-stage renal disease, MCNS: minimal change nephrotic syndrome, MAC: macroalbuminuria, MIC: microalbuminuria, NA: normoalbuminuria, NGAL: Neutrophil gelatinase-associated Lipocalin, TGF-β1: Transforming growth factor-beta, T1DM or T2DM: Type 1 or 2 diabetes mellitus, WT1: Wilm’s tumor-1.

## Data Availability

Not applicable.

## Abbreviations

AGE	Advanced glycation end products
BSA	Bovine serum albumin
CKD	Chronic kidney disease
CV	Cardiovascular
DKD	Diabetic kidney disease
DM	Diabetes mellitus
eGFR	Estimated glomerular filtration rate
ESRD	End stage renal disease
(u)EVs	(urinary) Extracellular vesicles
HbA1c	Glycated hemoglobin
HG	High glucose
GECs	Glomerular endothelial cells
GFB	Glomerular filtration barrier
GMCs	Glomerular mesangial cells
mi(cro)RNA	Micro ribonucleic acid
NTA	Nano particle tracking analysis
PS	phosphatidylserine
PTECs	Proximal tubular epithelial cells
ROC	Receiver operating characteristic
ROS	Reactive oxygen species
SEC	Size exclusion chromatography
TEM	Transmission electron microscopy
TF	Tissue factor
THP	Tamm Horsfall protein
UC	Ultra centrifugation
UF	Ultra filtration
